# Abdominal Pregnancy: Challenges in Recognition and Diagnosis in the Emergency Department

**DOI:** 10.7759/cureus.87627

**Published:** 2025-07-09

**Authors:** Saskah Thompson, Davin Powdhar, Saleem A Varachhia, Joanne Paul, Arvind Ramnarine

**Affiliations:** 1 Emergency Medicine, Eastern Regional Health Authority, Sangre Grande, TTO; 2 Faculty of Medical Sciences, The University of the West Indies, St. Augustine, TTO; 3 Pediatric Emergency Medicine, The University of the West Indies, St. Augustine, TTO; 4 Emergency Medicine, The University of the West Indies, St. Augustine, TTO

**Keywords:** abdominal pain in pregnant women, abdominal pregnancy, point-of-care-ultrasound, recognition barriers, misdiagnosis

## Abstract

An abdominal pregnancy (AP) is a unique type of ectopic pregnancy that occurs when the embryo implants within the peritoneal cavity. It is an obstetrical emergency that can be fatal to both the mother and the fetus. Being able to identify such an occurrence in the emergency department requires physicians to understand certain risk factors and clinical findings and appreciate diagnostic challenges. This report discusses the case of a young female who presented with abdominal pain and vaginal bleeding in what was believed to be an intrauterine pregnancy and later discovered to be an AP. It also highlights the differences in presentation of such cases as well as barriers that may impede diagnosis.

## Introduction

An abdominal pregnancy (AP) is a rare form of ectopic pregnancy that occurs when implantation occurs within the peritoneal cavity [1]. Though it occurs in 1 in 30,000 pregnancies worldwide, it is associated with a mortality rate as high as 20% for mothers and over 90% for the fetus [2,3]. APs are an obstetric emergency, one that should not be missed in an emergency department (ED), yet only an average of 20-40% are identified prior to surgery [4].

This report discusses the case of a young female patient who presented to the ED on consecutive occasions for abdominal pain and vaginal bleeding. However, due to varying reasons, her diagnosis of an intra-AP was only identified on a later visit. The case and discussion highlight the risk factors and differences in presentation of intra-AP versus intrauterine pregnancy. It also discusses barriers that exist in EDs that may cause this specific pathology to be unexpectedly missed.

## Case presentation

An 18-year-old primigravida Hispanic female presented to the ED complaining of abdominal pain and vaginal bleeding for one day. Via an interpreter, she reported that she was having sudden, sharp pain increasing in intensity in the supra-pubic area. The pain was currently a 4/10 in severity but increased with movement. She also reported mild vaginal bleeding that morning, with two sanitary napkins used and no clots noticed. Based on her last menstrual period, her period of gestation was calculated to be 11 weeks and 6 days. The patient had previously been seen in the ED for similar complaints three and four weeks ago. On both occasions, her urine pregnancy test was positive. On the second presentation, she reported feeling something moving in her abdomen. Each time, however, she left the ED before she could be assessed and examined by a physician.

She had not attended any clinic or received prenatal care prior to presentation. The patient had a past medical history significant for asthma; however, her last attack was several years ago, and she denied prior hospitalizations. She had no past surgical or significant gynecological history and no known allergies. She denied any alcohol or cigarette use. Her review of systems yielded no pertinent findings apart from the ones mentioned prior.

Her initial vitals were as follows: blood pressure of 125/68 mmHg, heart rate (HR) of 98 beats per minute (bpm), respiratory rate of 20 breaths per minute, temperature 36.2°C, SpO_2_ 99% on room air, and random blood sugar of 96 mg/dL. On physical examination, her abdomen was soft with supra-pubic tenderness and guarding. Bowel sounds were present. Speculum examination revealed scant blood in the vault with no active bleeding or clots noted. The cervical os was closed, and no excitation was appreciated. Her neurological exam was within normal limits.

An abdominal point-of-care ultrasound (POCUS) was performed in the ED, which showed a single fetus with cardiac activity and no obvious abnormalities. The transvaginal ultrasound probe was not available at the time of her assessment. Her urine analysis yielded +2 leukocytes. Based on her presentation and physical findings, a working diagnosis of threatened miscarriage was made, with a secondary diagnosis of a urinary tract infection in pregnancy. The patient was put on cardiac and blood pressure monitoring, with an intravenous access placed and relevant blood investigations ordered. Table 1 shows the patient's blood investigation results.

**Table 1:** Blood investigation results in the Emergency Department

Blood Investigations	Results/Units	Normal Range/Units
Hemoglobin	8.95 g/dL	13.8-17.2 g/dL
Mean corpuscular volume	85.6 fL	80-100 fL
White cell count	15.0 x 10^3^/uL	4.5-11 x 10^3^/uL
Platelets	249 x 10^3^/uL	150-400 x 10^3^/uL
C-reactive protein	19.55 mg/dL	0-5 mg/dL
Beta human chorionic gonadotropin	64,404 mlU/mL	<5 mlU/mL

**Figure 1:**
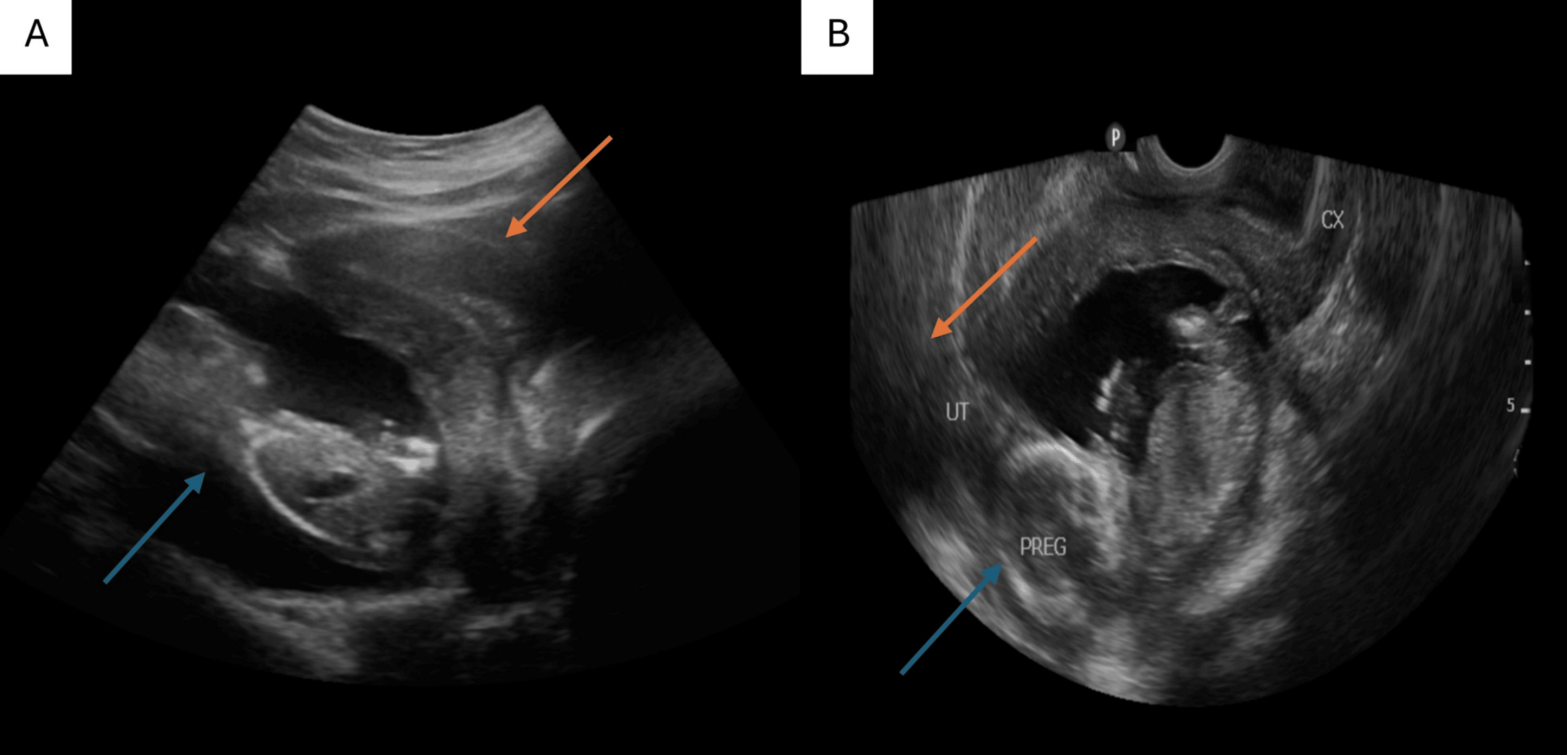
Obstetric ultrasound. Still images (A and B) show an empty uterus (orange arrow) and the fetus located outside the uterus (blue arrows).

A urine sample was sent for culture, and a request for review by the obstetric team on call was made. The patient was started on 1 liter of normal saline and given acetaminophen for analgesia. She was subsequently reviewed by the obstetric team who also conducted a bedside ultrasound, confirming a single intrauterine gestation with a posteriorly located placenta and normal liquor volume. The patient was kept for observation with the same working diagnosis as above. An official ultrasound was requested and obtained a few hours later. However, this scan revealed an empty uterus and an AP with a live fetus (HR 146 bpm), with parameters giving an estimated gestational age of 15 weeks and 3 days (Figure 1).

**Figure 2:**
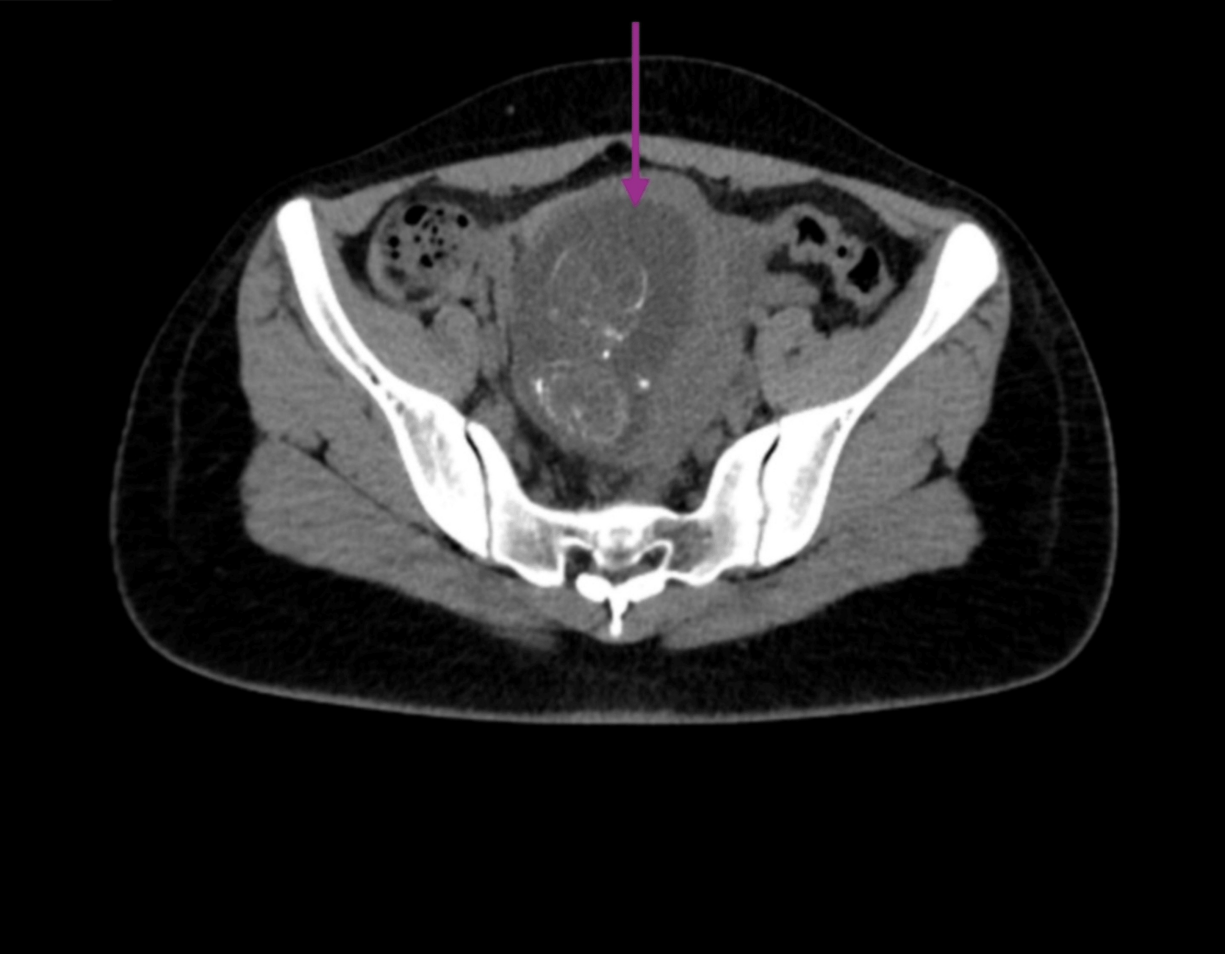
Computed tomography scan confirming extrauterine pregnancy (purple arrow)

The obstetric team reassessed the patient, and as the patient was still hemodynamically stable, she was sent for a computed tomography (CT) scan, as magnetic resonance imaging (MRI) was unavailable. This also confirmed an extrauterine pregnancy, with the amniotic cavity containing the formed fetus and amniotic fluid. The placenta was noted to be lying to the left of the amniotic cavity (left pelvis) and posteriorly situated. There was no evidence of implantation of the placenta into the adjacent vascular structures or pelvic viscera, and no free fluid was appreciated. CT findings can be seen in Figure 2.

A multi-disciplinary team discussion between obstetrics and general surgery was held, with the patient and her relatives being counseled on the need for urgent surgery, as well as the associated risks and loss of the fetus. Consent was obtained for an exploratory laparotomy, and during the surgery, the fetus and placenta were removed. Left salpingo-oophorectomy was also performed due to the pregnancy being adhered to the left ovary. Her estimated blood loss was 1.3 liters. She did favorably post-operation. She had a postoperative hemoglobin level of 8.26 g/dL and was transfused with one unit of packed red blood cells. She was discharged a few days later with wound review, gynecology clinic follow-up, and social worker follow-up as an outpatient.

## Discussion

AP presentations are unique as they are among the least likely locations for embryo implantation. They make up less than 2% of possible sites for ectopic pregnancies, with pregnancies in the ampulla being most common (80%), followed by isthmic (12%), fimbrial (5%), cornual (2%), and ovarian and cervical (0.2%) [5]. APs are seen in higher incidences in low- and middle-income countries [6,7]. This has been attributed to a lack of patient education and a lack of accessible and adequate medical care [6]. Conversely, there has also been a notable rise in APs in developed countries in recent years due to a significant increase in the development and demand for assisted reproductive technology (ART) [8].

There is still much to be understood about this condition, and considerable information has mostly been garnered from published case reports over the years. AP can be categorized into two types: primary and secondary. A primary AP occurs when the fertilized egg deposits directly into the abdominal cavity, while a secondary AP is when the embryo is implanted elsewhere in the uterine cavity but later migrates to the abdomen [6]. In 1942, a criteria known as Studdiford's criteria was created to define the conditions under which an AP can be confirmed as primary. The three requirements are normal fallopian tubes and ovaries, no findings of utero-peritoneal fistula, and a pregnancy localized to the abdominal cavity [9]. This can include a location within solid organs, such as the liver or spleen, or the omentum, with the most common site being the recto-uterine pouch [10]. Based on her intra-operative findings, our patient likely had a secondary AP, where the embryo migrated from the left ovary.

Due to the high mortality rates of APs, it is important for emergency physicians to be able to correctly identify patients who may be at risk for such occurrences. Some risk factors for AP are the same as those for ectopic pregnancies. These include women with pelvic inflammatory disease, endometriosis, a history of ART, ectopic pregnancy, tubal surgery, or scarring [11]. According to a case report by Mpogoro et al., a primary AP occurring in the absence of ART or history of uterine scarring is uncommon, and, as a result, most cases are often secondary [12].

Another factor that attributes to AP being missed is that it lacks a unique or atypical presentation. Firstly, APs can be asymptomatic. Symptoms, when present, are usually mistaken for other more commonly presenting gynecological ailments. Patients may report abdominal/pelvic pain with fetal movements, vaginal bleeding, anemia, gastrointestinal upset, a hard, long fixed cervix, and a palpable abdominal mass [10,13]. The patient in this case report had three of these findings: normocytic anemia, menorrhagia, and abdominal pain. The most common symptoms of APs are abdominal pain and vaginal bleeding, which are symptoms that are often mistaken for a miscarriage [13].

Another factor contributing to missed recognition of AP in the ED is ultrasound user variability. POCUS is often used as the primary diagnostic modality for women presenting with abdominal pain or gynecological complaints in an ED. Yet, many APs can often be missed on ultrasound [14]. In the case of this patient, her initial radiological examination was an abdominal ultrasound, which showed the fetus with cardiac activity and fetal movements, thought to be an intrauterine pregnancy. Though ultrasound plays an important role in the diagnosis of an ectopic pregnancy, according to Baffoe et al., it can be unreliable in later stages of gestation, even on repeat scans [2]. This is due to the larger gestational sacs appearing as being intrauterine. Additionally, in China, Chen et al. found in a case series review of 17 patients with AP that 84.6% of patients had APs that were missed on initial imaging [4].

Ideally, a transvaginal ultrasound is the test of choice for the diagnosis of an ectopic pregnancy as it has a higher sensitivity (up to 99%), allowing for better views of the uterine cavity and adnexa; however, this type of ultrasound is not often performed in the acute ED setting [10]. Other imaging tests for AP include CT and MRI, which can also aid in diagnosis when the exact location or implantation of the placenta is unclear [6,10]. MRI is safe for both the mother and the fetus and can help guide obstetrician surgical treatment approaches.

In this particular case, it is also important to consider non-medical and social factors that could have led to this condition not being identified sooner. English was not this patient’s native language, and medical terminology can often be lost in translation between patients and their health care providers. Race, age, socioeconomic status, and patient education all also play important roles in adherence to medical therapy. A cross-sectional study by Albarrak et al. including 71 patients in Saudi Arabia found a significant correlation between patient understanding and compliance (p=0.05) [15]. Diaouga et al. in their case report also highlighted a higher incidence of APs in developing countries due to a lack of patient adherence and follow-up [16]. Furthermore, an extensive literature review by Jin et al. including 102 articles identified African Americans and Hispanics to be among the minority groups with poor patient compliance [17]. This was similarly seen in patients under the age of 40 years [17]. These are very pertinent factors, as they highlight a subset of women in whom APs are more likely to be missed, such as the patient in this case report who fell into more than one of these categories.

The complications from AP can be local and systemic. They range from fistula and abscess formation to bowel obstruction, renal failure, sepsis, disseminated intravascular coagulation, hemorrhagic shock, and death [7,18]. These are all life-threatening but preventable conditions once diagnosis is made in a timely manner. Our patient had repeated episodes of presentation to the ED but always left before an ultrasound or proper assessment could have been conducted due to long wait times and personal obligations. It is pertinent that the severity of APs, complications, and need for early identification be stressed to patients like her, especially in cases where social barriers or impedances exist.

It must be mentioned that expectant management of an AP is a possibility. There have been cases where in the setting of an advanced AP, the fetus was carefully monitored and successfully delivered [2,18,19]. Many infants in these cases, however, tend to be born with chromosomal abnormalities or have a higher likelihood of birth defects and post-natal complications [2,20].

It is likely that due to the rise in delayed motherhood, there may be increased utilization of ART and, as a result, more presentations of APs in the ED. It would be beneficial for ED physicians to always have AP in their list of differentials especially for advanced pregnancies that have poor antenatal care. Additionally, physicians should also be mindful of the possible risk factors, red-flag symptoms, and diagnostic variability that can exist for this subset of patients. Furthermore, the key role of patient understanding and education in compliance should be remembered and not taken for granted.

Prevention strategies that can be adopted in EDs involve, firstly, early identification and management of gynecological risk factors for ectopic pregnancies, secondly, focusing on patient education and awareness of ectopic pregnancies and APs as a subset and how symptoms may present, and, finally, improving and developing diagnostic techniques among physicians. These considerations can help accelerate timely identification of life-threatening gynecological pathologies and reduce prolonged waiting times. If all of the above factors are considered and appropriate clinical approaches are applied, there can be earlier and timely recognition of AP patients.

## Conclusions

Due to variations in patient presentation, physical findings, and ultrasound interpretation, APs will unfortunately continue to be a challenging diagnosis. ED physicians need to consider red flags and be vigilant in the overlap of patient presentations and symptomologies. Consideration must also be given in cases of ultrasound ambiguity, ensuring the utilization of other imaging modalities such as MRI if fetal or placenta location is unclear. Additionally, focus needs to be placed on strengthening ED doctors' skills in transvaginal ultrasound.

Currently, there are no established criteria for detecting APs. Hence, a higher index of suspicion is needed especially when dealing with atypical presentations or when notable barriers exist. Ideally, established guidelines for the detection of AP should be developed and applied in the Caribbean and developing countries, where early recognition is of significant importance.
